# Molecularly guided pharmacotherapy for obesity: emerging targets, clinical efficacy, and personalized therapeutic approaches

**DOI:** 10.3389/fphar.2026.1988828

**Published:** 2026-10-22

**Authors:** Jianchao Mao, Rong Hu

**Affiliations:** The Department of Endocrinology, Yuyao Fourth People’s Hospital, Ningbo, Zhejiang, China

**Keywords:** genetic determinants, GLP-1 receptor, incretin therapy, molecular targets, multi-agonists, obesity, pharmacotherapy, therapeutic response

## Abstract

Obesity is a complex, chronic disease whose aetiology is complex and highly variable, and is linked to a network of neural, endocrine, adipose, genetic, epigenetic and metabolic pathways. This molecular complexity also drives significant differences in the clinical course of disease, drug sensitivity and weight regain, thus making standardized treatment, which is largely based on BMI and clinical empiricism, challenging. This narrative review explores the molecular mechanisms involved in obesity and the potential to apply the knowledge of molecular mechanisms to develop more personalized treatment strategies using advances in pharmacology. It focuses on hypothalamic appetite-satiety pathways, gut–brain hormonal communication, inflammation and metabolic dysfunction in adipose tissue, and known and novel drug targets. The review focuses on the evidence available on GLP-1 receptor agonism, GIP–GLP-1 co-agonism, multi-agonism with glucagon, signaling of amylin, melanocortin pathways, and novel central and peripheral targets. Studies have shown that some dual- and triple-receptor drugs are more effective at causing weight loss, and practitioners should consider tolerability, sustainability, cardiometabolic effects, and response when choosing drugs or drug combinations for patients. Inter-individual variability may be further explained by genetic, epigenetic, metabolic, and tissue-specific factors, which may help identify mechanisms of resistance. In conclusion, Inter-individual variability may be attributed to genetic, epigenetic, metabolic, and tissue-specific factors, and most suggested molecular or biomarker predictors have not yet proven sufficiently valid or useful for routine drug selection. Receptor-targeted drugs, including GLP-1 receptor agonists and GIP–GLP-1 co-agonists, are currently clinically actionable for patients with common obesity and available to specific patients with obesity, genotype-directed molecular therapeutic drugs are clinically actionable only for selected genetically defined types of obesity. However, the use of pharmacogenomic, biomarker, and multi-omics methods to guide the choice of anti-obesity drugs are still in their early stages of clinical investigation.

## 1 Introduction

Obesity is not just a single disease, it is a complex, chronic condition. This complexity arises from interactions among several biological and environmental factors: hypothalamic and brainstem neural circuits involved in appetite and satiety, gastrointestinal and endocrine signals, adipose tissue biology, genetic susceptibility, epigenetic regulation, metabolic dysfunction, diet, physical activity, sleep, psychosocial factors, medications, and the environment. These play out in several physiological systems and may give rise to various obesity phenotypes, metabolic complications, treatment responses, and weight-regain-prone phenotype. The differences in body composition and/or adipose-tissue function could also contribute to the wide variability in metabolic parameters and treatment responses observed in people with the same body mass index (BMI). Adipose-tissue composition and function has been shown to provide biological information beyond adiposity in recent cellular studies that identified distinct populations of adipose-tissue cells corresponding to metabolic health (Reinisch et al., 2025). Multi-omics and machine-learning studies of genetic differences between obese participants have also revealed interconnected molecular pathways, consistent with the notion that obesity is a biologically heterogeneous disorder rather than a single molecular entity (Li et al., 2025a). Integrated proteomic and metabolomic profiling has also identified obesity-associated molecular signatures across several metabolic pathways, as well as potential therapeutic targets (Li et al., 2025b).

This complexity lies not only in the number of adipocytes but also in their function and metabolic communication. Changes in adipose cellular composition and transcriptomic signatures are linked to glycemic dysfunction, further highlighting the role of adipose tissue-specific molecular remodelling in obesity-related metabolic abnormalities (Paulí et al., 2025). Furthermore, even after significant weight loss, obesity can have long-term biological impacts. Evidence that obesogenic molecular adaptations are epigenetic in adipose tissue highlights the potential of an epigenetic memory of obesity and vulnerability to weight regain (Hinte et al., 2024). At the central level, newly identified hypothalamic neuronal populations add to the molecular architecture regulating appetite and energy intake, with obesity regulated by distributed networks rather than a single defective pathway (Tan et al., 2024).

With the rising availability of mechanism-based pharmacotherapies, the question that may be raised is whether molecular heterogeneity can underlie more individualized treatment strategies. Treatment effects also depend on genetic makeup, the integrity of key signalling pathways, metabolic phenotype, and tissue-specific responses. Evidence from recent multi-ancestry studies indicates that the genetic factors identified so far may play only a minor role in variability in weight loss with GLP-1 receptor agonists, while other genetic measures, such as BMI polygenetic scores, have been associated with weight loss after bariatric surgery (German et al., 2025). Importantly, the molecular defect that is being targeted will also impact pharmacological efficacy. Pharmacological responsiveness, however, may be maintained despite impaired physiological function, since weight loss with tirzepatide was significant in people with pathogenic MC4R mutations, suggesting that the pharmacological pathway may be sufficient even in the absence of a physiological pathway (Bhatnagar et al., 2025). Other peripheral-level signatures of adipogenesis and lipid mobilisation identified in experiments further support the idea that targeting molecular processes in adipose tissue may alter adipogenesis and lipid mobilisation (Xu et al., 2025).

Obesity is an important and growing public-health problem in China. The trend of adults becoming overweight and obese in China has been well recorded in national epidemiology surveys which have found prevalence rates to be affected by age, sex, geographic location and the thresholds used for determining overweight and obesity. The prevalence of overweight and obesity is high and increasing, and this set the backdrop for more effective and personalized strategies to tackle obesity at a regional level (Wang et al., 2021).

These advances support molecularly informed pharmacologic management of obesity, integrating therapeutic targets, mechanisms of action, the biological characteristics of obesity, and factors determining response to therapy. To summarize, molecularly guided pharmacotherapy is an umbrella term for five classes of translational relevance: (1) molecular mechanisms of drug action, which describe the way drugs interact with specific receptors and signaling pathways; (2) disease-associated biomarkers, which represent a characterization of the biological heterogeneity but do not necessarily predict treatment response; (3) predictive biomarkers, which might identify differences in therapeutic efficacy, tolerability, or resistance; (4) validated genotype-directed therapy, where a specific molecular defect directly influences the therapeutic choice; and (5) investigational molecular and multi-omics-based strategies, which aim to discover new therapeutic targets or novel therapeutic responsive phenotypes, but are still under clinical development. These categories do not necessarily overlap and vary in their clinical actionability. Thus, this review focuses on molecular mechanisms and therapeutic targets, the difference between biological biomarkers and predictive markers, genetically defined therapeutic strategies, and emerging multi-omics approaches, depending on their current translational status. The purpose of this framework is to define the role that molecular understanding can play in the selection of treatment, understanding of treatment resistance and in the development of more personalized treatment with pharmacotherapy for obesity.

### 1.1 Narrative review methodology

The narrative review used a structured search of PubMed, Scopus, Web of Science, and Google Scholar, with the final literature search conducted between 2016 and 2026. Combinations of keywords, such as obesity, molecular mechanisms, pharmacotherapy, drug targets, incretin signalling, adipose biology, genetic determinants, biomarkers, precision medicine and personalised treatment were incorporated into searches. The focus was on recent original research, mechanistic studies, clinical trials, and translational research with foundational studies included where required to support established biological pathways. The literature was critically summarized with reference to the molecular mechanisms, pharmacological targets, clinical efficacy, determinants of therapeutic response and emerging personalized treatment strategies. The literature identified through the search was also used to contextualize the current regulatory status of established and investigational pharmacotherapies and emerging molecularly guided approaches.

## 2 Molecular regulation of energy homeostasis and obesity

Obesity results from complex dysregulation of biological systems that control energy intake, energy expenditure, and metabolic homeostasis. Central hypothalamic circuits integrate hormones and nutrition to regulate appetite and satiety, and gut–brain communication regulates gastrointestinal hormones and feeding and metabolic neural pathways. Overdistention of adipose tissue at the periphery may lead to inflammation, adipocyte dysfunction, insulin receptoresistance and a variety of metabolic abnormalities. These interrelated mechanisms provide a molecular explanation for the multifactorial nature of obesity and the multiple biological pathways targeted by drugs to control appetite, hormonal signalling, adipose tissue dysfunction, and metabolic pathways.

### 2.1 Hypothalamic appetite and satiety circuits

The hypothalamus is a key regulator of energy homeostasis and integrates hormonal, nutritional, and neural signals to regulate appetite, satiety, energy expenditure, and body weight. In the arcuate nucleus (ARC), orexigenic neurons (NPY/AgRP) activate mechanisms that increase food intake, while Proopiomelanocortin (POMC) neurons turn off food intake via α-MSH (Melanocyte-Stimulating Hormone) and activation of MC4R (Melanocortin-4 receptor). These neuronal pathways interact with peripheral signals such as leptin, insulin, and gut-derived hormones, and disruption of this finely tuned network can increase hunger and energy intake, which in turn can lead to obesity (Gan et al., 2024). Altered hypothalamic regulation may also maintain excess adiposity and weight regain by biologically protecting a higher body weight. This helps to recognise obesity as a chronic disease, not merely a result of individual eating habits (Purnell and le Roux, 2024). These central and peripheral signals enable the hypothalamus to constantly fine-tune feeding behaviour, based on energy availability and energy needs. Therefore, disruptions in any of these circuits may be an important therapeutic target for re-establishing long-term control of appetite and weight loss.

### 2.2 Gut–brain signalling and hormonal regulation

The gut–brain axis is a highly integrated communication system mediated by gastrointestinal hormones, neural pathways, and metabolic signals that control appetite, satiety, food intake, and energy expenditure. After eating, enteroendocrine cells secrete hormones such as glucagon-like peptide-1 (GLP-1), glucose-dependent insulinotropic polypeptide (GIP), peptide YY (PYY), cholecystokinin (CCK), and oxyntomodulin. At the same time, ghrelin is mainly an orexigenic signal during fasting. These hormones act internally via circulating blood and vagal afferents (nerves that carry signals to and from the brain) and affect hypothalamic and brainstem circuits that regulate hunger and satiety. These signals coordinate and help synchronise food intake with nutritional needs and control the postprandial metabolic response. Gut hormonal signals can affect appetite regulation and energy balance, making these pathways key molecular targets for pharmacologic obesity treatment. Gut hormones play crucial roles in regulating appetite, with anorexigenic hormones suppressing appetite and affecting metabolic homeostasis (Hong and Choi, 2024).

Therapeutic implications of gut–brain hormonal interactions are not confined to any particular hormone pathway, as multiple signals share common neural and endocrine pathways. These hormones, including GLP-1, GIP, PYY, CCK, and others, can modulate neurons in the hypothalamus and brainstem, signal through the vagus nerve, affect gastric emptying, insulin secretion, and satiety, and thus could represent several points for pharmacological intervention. Roh and Choi (Roh and Choi, 2023) highlighted the importance of the mechanistic basis for current and emerging anti-obesity therapies that manipulate hormonal gut–brain signalling, especially those aimed at mimicking or augmenting endogenous satiety signals. These hormonal signals converge centrally, acting on neural pathways involved in feeding behaviour and energy expenditure, and peripherally, where they influence glucose and lipid metabolism. Gut-hormone regulation of metabolism is linked to the brain via interconnected pathways involving the hypothalamus, brainstem, and vagal system (Liu et al., 2025). This integrated biology supports novel therapeutic strategies based on individual hormonal receptors and complementary hormone receptors to improve appetite suppression, metabolic benefits, and sustained weight loss, which could inform molecularly guided pharmacotherapy.

### 2.3 Adipose tissue, inflammation, and metabolic dysfunction

Adipose tissue is a dynamic metabolic and endocrine organ which controls energy storage, glucose and lipid metabolism, and systemic metabolic homeostasis. Adipose tissue consists of distinct functional depots: white adipose tissue (WAT) stores excess energy as triglycerides, while brown adipose tissue (BAT) has a highly active, mitochondria-supported metabolism and can support thermogenesis. WAT can increase in size by increasing adipocyte size (hypertrophy) and number (hyperplasia), and overexpanded adipocytes are linked to inflammatory and metabolic dysfunction, whereas BAT is linked to energy dissipation and glucose and lipid metabolism. These functional differences make adipose tissue a key molecular target for obesity and for developing obesity-targeting drugs that affect energy storage and expenditure. In obesity, excess nutrient storage increases inflammatory mediators, disrupting metabolic signalling pathways and causing adipocyte hypertrophy and dysfunction. The relationship between adipocytes, immune cells, and inflammatory pathways can lead to insulin resistance and influence adipokine secretion, as well as other cardiometabolic abnormalities (Xu et al., 2024).

The balance between inflammatory activation and resolution also matters in persistent low-grade inflammation, and insufficient resolution may sustain adipose tissue dysfunction and metabolic abnormalities (Soták et al., 2025). As such, these processes are also relevant to molecularly guided obesity pharmacotherapy, as targeting adipose inflammation, immune–metabolic interactions, and tissue remodelling could complement gut hormone signalling and appetite-control therapies primarily focused on these processes. Understanding these mechanisms could provide a basis for discovering other therapeutic targets to treat both excess adiposity and its metabolic sequelae. These mechanisms provide the biological basis for therapeutic targeting and should be distinguished from molecular markers validated for patient-specific treatment selection. Figure 1 illustrates the integration of central and peripheral signals regulating energy homeostasis and their major pharmacological targets.

**FIGURE 1:**
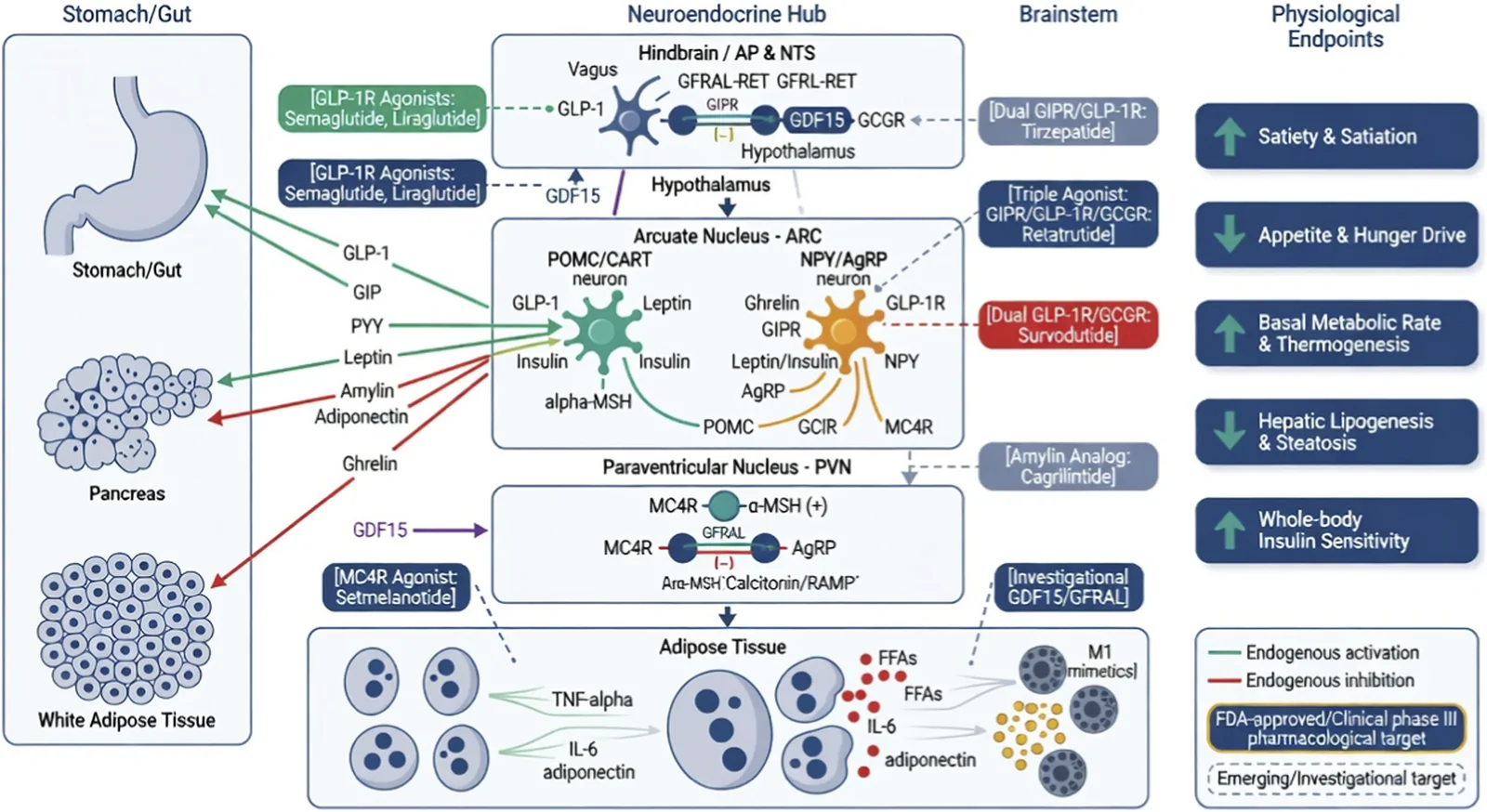
Molecular regulation of energy homeostasis and pharmacological targets. The figure created by the authors based on published literature summarizes the major peripheral, gut–brain, hypothalamic, and adipose-tissue pathways involved in energy homeostasis and their pharmacological targets.

## 3 Established and emerging molecular targets for obesity pharmacotherapy

Drugs used to treat obesity have evolved from simply suppressing appetite to targeted modulation of pathways that control energy intake, energy expenditure, glucose and lipid metabolism, adipogenesis, inflammation, and metabolic homeostasis. Several molecular targets and signalling pathways regulate appetite, energy expenditure, glucose metabolism, and body weight. These pathways provide the biological rationale for existing obesity treatments and for new drugs targeting multiple metabolic pathways. Established targets (e.g., GLP-1, melanocortin signalling) have proven clinically relevant, and emerging central and peripheral targets offer potential for novel combination therapies and more individualised treatment strategies. Identifying these molecular targets will be essential for developing drugs that address the biological diversity of obesity and ultimately support better, longer-lasting, and possibly individualised weight management (Elizalde-Romero et al., 2024).

The molecular targets referred to in this section are mainly mechanisms of drug action. Although the molecular mechanisms of receptor binding and subsequent signaling dictate the effects of pharmacological agents on appetite, energy expenditure, glucose metabolism and body weight, the mere identification of a molecular target does not imply that the molecular profile of a patient can be used to guide the selection of that target. The selection of molecular treatments that may be specific to the patient is addressed separately in other sections.

### 3.1 GLP-1 receptor signalling

The GLP-1 receptor pathway is a key pathway for controlling appetite and satiety, glucose metabolism, and body weight. GLP-1 receptor activation inhibits food intake, slows gastric emptying, increases insulin release in the presence of glucose, and decreases glucagon release, all of which help maintain metabolic homeostasis and control appetite. The physiological role of GLP-1 signalling and its clinical implications for chronic weight management are well established and are being translated into incretin-based therapies (Holst, 2024). Pharmacological GLP-1 receptor agonists prolong activation of this pathway and can cause significant weight loss, establishing them as a key therapeutic target for obesity. In addition to their clinical effectiveness, multi-receptor drugs have been developed that harness both GLP-1 activity and other metabolic pathways, enabling more targeted obesity treatment strategies (Myerson and Paparodis, 2024).

### 3.2 GIP–GLP-1 receptor co-agonism

GIP–GLP-1 receptor co-agonism is an exciting new development in molecularly targeted obesity drugs that activates both the GIP and GLP-1 receptors simultaneously. Combined activation of the GIP and GLP-1 receptors may enhance glucose-dependent insulin secretion, glucagon regulation, appetite reduction, and metabolic homeostasis beyond selective GLP-1 receptor activation alone, potentially leading to greater weight loss. The complementary mechanisms of these receptors offer a molecular basis for using dual agonists like tirzepatide to broaden the therapeutic applications of incretin-based strategies for obesity (Liu, 2024). Additionally, evidence suggests that GIP receptor activation plays a significant role in tirzepatide’s overall pharmacologic action, rather than being a nonessential part of GLP-1 receptor activity. This supports the idea that simultaneous activation of multiple metabolic receptors can increase treatment efficacy and is an important step toward more advanced, molecularly targeted obesity therapy (Gasbjerg et al., 2023).

### 3.3 Glucagon-based multi-agonism

Glucagon-based multi-agonism could benefit obesity treatment by activating the glucagon receptor alongside GLP-1 signalling to suppress appetite and affect metabolism. Glucagon’s ability to stimulate energy expenditure, lipid utilisation, and hepatic metabolic activity may support weight loss, and combining glucagon activity with GLP-1 receptor activity could be even more effective for weight loss and improve metabolic control. Glucagon agonism has shown promise in combination therapies for obesity and other metabolic disorders (Winther and Holst, 2024). In addition, gastrointestinal side effects and the need for longer-term safety data are pertinent considerations, as GLP-1/glucagon receptor dual agonists have shown positive weight-loss outcomes and metabolic effects in clinical trials. In contrast, safety data remain pending. This complementary approach to receptor-based therapy is a promising avenue for studying molecularly targeted therapies for obesity (Deng et al., 2024). Multi-agonists can have complementary effects by targeting multiple metabolic pathways simultaneously and can have greater therapeutic potency than single-receptor agonism in certain contexts. The potential benefit of receptor co-agonism in tolerability and treatment resistance is unclear and needs confirmation in clinical studies. Expansion of receptor complexity, therefore, should be regarded as a possible way to improve therapeutic effects, not as a sign of decreased adverse effects and drug resistance.

### 3.4 Amylin and amylin-receptor pathways

The amylin receptor pathway is a novel target for obesity drugs because amylin is a pancreatic hormone that regulates energy intake and postprandial satiety. Activation of amylin receptors triggers a sense of fullness and may decrease food consumption, and pharmaceutical amylin analogues could provide a prolonged effect to control food intake and body weight. In adults with overweight or obesity, clinical evidence showed cagrilintide, a long-acting amylin analogue, has potential to induce weight loss, suggesting the relevance of this pathway (Lau et al., 2021). More recently, however, amylin analogues have attracted growing interest because they can be used as combination therapies for weight reduction, as well as standalone therapies, where their complementary effect may improve weight reduction over conventional therapies (Panou et al., 2024). Therefore, amylin-receptor signalling is a potentially interesting molecular target in obesity pharmacology for expanding and personalising obesity drugs.

### 3.5 Melanocortin–MC4R signalling

The melanocortin pathway controls feeding behaviour, satiety, and energy balance, and melanocortin-4 receptor (MC4R) signalling is a key molecular target in genetically defined obesity. The melanocortin peptides inhibit food intake and increase satiety by binding to the MC4R pathway and disruption of this pathway can lead to hyperphagia and obesity. Structural studies have also uncovered the molecular mechanism of MC4R activation through ligand binding and the initiation of cellular signalling pathways that promote satiation, helping elucidate the mechanism behind MC4R-based therapeutics (Israeli et al., 2021). Setmelanotide is an MC4R agonist that has been shown to be effective in causing significant weight loss in patients with severe obesity caused by either POMC or LEPR deficiency (Clément et al., 2020; Markham, 2021) Under these circumstances, the molecular defect is upstream of MC4R, so the drug can stimulate an intact MC4R downstream of the molecular defect. Pathogenic variants of the receptor targeted by setmelanotide, however, have a direct effect on the receptor. As such, a mutation in MC4R should not be used as a clear-cut criterion for setmelanotide treatment. These variations emphasize the need to account for the site of molecular defects in genetically defined obesity when targeting drugs.

### 3.6 Emerging central and peripheral targets

New obesity treatments increasingly target the many central and peripheral pathways that regulate hunger/satiety, energy expenditure, and metabolic homeostasis. Beyond selective GLP-1 receptor activation, dual GLP-1/GIP and GLP-1/glucagon receptor signalling may also affect neural circuits involved in appetite regulation, potentially augmenting weight-loss and metabolism-beneficial effects. These complementary pathways act at the hormonal level and target multiple aspects of energy regulation rather than relying on a single molecular target (D'Ávila et al., 2024). As knowledge of these pathways grows, more opportunities emerge to develop broader-acting, longer-lasting pharmacotherapies for obesity.

The therapeutic pipeline is also expanding beyond the traditional GLP-1 receptor agonists to novel incretin combinations and other hormonal and metabolic targets. These approaches may offer alternatives for people who are not responding well, are not tolerating other treatments, or are regaining weight on other treatments. New pharmacological combinations with complementary mechanisms could improve therapeutic response and allow optimisation of treatment based on the metabolic characteristics and responses of the individual being treated (Sztanek et al., 2024). This variety of molecular targets is a significant step toward targeted, individualised pharmacotherapy for obesity. The major established and emerging molecular targets currently being investigated for obesity pharmacotherapy, together with their physiological roles, pharmacological approaches, and developmental status, are summarized in Table 1.

**TABLE 1:** Established and emerging molecular targets for obesity pharmacotherapy.

Molecular target/pathway	Physiological and pharmacological role	Representative pharmacological approach	Major therapeutic effects	Development/status	References
GLP-1 receptor (GLP-1R)	Enhances satiety, reduces food intake, delays gastric emptying, and improves glucose regulation	GLP-1 receptor agonists	Weight reduction, improved glycemic control and cardiometabolic risk	Established	(Holst, 2024; Wilding et al., 2021)
GIP receptor (GIPR) + GLP-1R	Combines incretin signaling with complementary effects on appetite, insulin action, and energy metabolism	Tirzepatide and other dual agonists	Greater weight reduction and metabolic improvement than single-pathway incretin therapy	Established clinical therapy	(Gasbjerg et al., 2023; Jastreboff et al., 2022)
Glucagon receptor (GCGR) + GLP-1R	GLP-1-mediated appetite suppression combined with glucagon-mediated effects on energy expenditure and lipid metabolism	Survodutide and other GLP-1/glucagon co-agonists	Weight loss, increased energy expenditure and metabolic effects	Investigational, Clinical development	(Winther and Holst, 2024; le Roux et al., 2024)
GLP-1R + GIPR + GCGR	Simultaneous modulation of appetite, insulin action, energy utilization and energy expenditure	Retatrutide	Up to 24.2% mean weight reduction at 48 weeks in the highest-dose group and broad metabolic effects	Investigational; phase 2/ongoing Clinical development	(Jastreboff et al., 2023)
Amylin receptor	Promotes satiety, reduces food intake and contributes to postprandial metabolic regulation	Cagrilintide and other amylin analogues	Appetite suppression and Dose-dependent weight reduction; up to 10.8% at 26 weeks in the phase 2 trial	Investigational	(Lau et al., 2021; Panou et al., 2024)
GLP-1R + amylin receptor	Combines incretin-mediated appetite regulation with amylin-mediated satiety	Amycretin	Up to 24.3% mean weight reduction at week 36 in a phase 1b/2a study	Early clinical development	(Dahl et al., 2025)
Melanocortin-4 receptor *(MC4R)*	Central regulator of satiety and energy balance downstream of *POMC* signaling	Setmelanotide for selected genetically defined/syndromic or acquired hypothalamic obesity	Marked weight reduction in selected genetic forms of obesity	Established genotype-/syndrome-specific therapy	(Clément et al., 2020; Haqq et al., 2022; U.S. Food and Drug Administration, 2024; European Medicines Agency, 2026)
*MC4R* pathway (acquired hypothalamic obesity)	Hypothalamic injury or impairment disrupts *MC4R*-pathway signalling; not a genotype-defined condition	Setmelanotide in acquired hypothalamic obesity (age ≥4 years)	Reduction in BMI and hunger	Approved (FDA, EC)	(Roth et al., 2024; U.S. Food and Drug Administration, 2024; European Medicines Agency, 2026)
*GDF15–GFRAL* pathway	Regulates appetite and energy expenditure through hindbrain signaling	*GDF15/GFRAL* pathway modulation	Potential appetite suppression and metabolic effects; clinical utility remains investigational	Experimental	(Yang et al., 2017)
Adipose-specific molecular pathways	Regulate adipogenesis, lipolysis and adipose metabolic function	Emerging molecular targets	Potential modification of adipose dysfunction and lipid handling; patient-selection utility remains unvalidated	Preclinical/experimental	(Xu et al., 2025)

## 4 Pharmacological mechanisms and clinical evidence of established and emerging therapies

There are several medications used for obesity that work on various mechanisms to reduce appetite, enhance satiety, optimise metabolic function and promote weight loss. The clinical efficacy, safety, and tolerability of these treatments can differ, as can their impact on cardiometabolic outcomes and the sustainability of treatment benefits; clinicians should consider these factors when choosing a treatment. Several newly developed incretin therapies, such as GLP-1 receptor agonists and dual- and triple-receptor agonists, have proven highly effective for weight loss in clinical trials, and other approved drugs still have a role as clinically important weight-loss treatments. Importantly, the therapies mentioned in this section are not all at the same stage of regulatory and developmental stage. The status of each agent is based on the United States Food and Drug Administration (FDA). Each agent is designated as approved for chronic weight management, approved for a genetically defined obesity, approved for another medical indication (not obesity), or still in the investigational stage for obesity. Tables 1 and 2 include this distinction for individual agents, as regulatory approval and routine clinical availability may differ.

**TABLE 2:** Pharmacological mechanisms, clinical efficacy, and safety considerations of anti-obesity therapies.

Therapy	Molecular target/mechanism	Key clinical evidence	Reported weight-loss efficacy	Major benefits	Clinical status/actionability	Important limitations/safety considerations	References
Semaglutide 2.4 mg	GLP-1R agonism; reduces appetite and food intake	STEP 1 randomized trial	14.9% mean weight reduction at 68 weeks	Significant weight loss; cardiometabolic improvement	Established clinical therapy	Predominantly gastrointestinal adverse effects; chronic treatment generally required	(Wilding et al., 2021)
Tirzepatide	GIPR + GLP-1R agonism	SURMOUNT-1 randomized trial	15.0%–20.9% mean weight reduction at 72 weeks, depending on dose	FDA-approved for chronic weight management, and metabolic improvement	Established clinical therapy	Gastrointestinal adverse effects; long-term treatment required	(Jastreboff et al., 2022)
Retatrutide	GLP-1R + GIPR + GCGR agonism	Phase 2 randomized trial	Up to 24.2% mean weight reduction at 48 weeks	Weight loss with effects on multiple metabolic pathways	Investigational	Gastrointestinal adverse effects and need for longer-term safety data	(Jastreboff et al., 2023)
Survodutide	GLP-1R + GCGR agonism	Phase 2 efficacy trial/phase 3 development	Dose-dependent weight reduction; 14.9% at 46 weeks with the 4.8-mg dose in the phase 2 trial	Combines appetite suppression with glucagon-mediated metabolic effects	Investigational	Gastrointestinal adverse effects	(le Roux et al., 2024)
Cagrilintide	Long-acting amylin receptor agonism	Phase 2 randomized trial	6.0%–10.8% mean weight reduction at 26 weeks, depending on dose	Complementary satiety mechanism; potential combination partner	Investigational	Gastrointestinal tolerability	(Lau et al., 2021)
Amycretin	GLP-1R + amylin receptor agonism	Phase 1b/2a randomized controlled study	Up to 24.3% mean weight reduction at week 36 in a phase 1b/2a study	Dual appetite-regulatory mechanism	Investigational/early clinical development	Early development; longer-term efficacy and safety remain uncertain	(Dahl et al., 2025)
Setmelanotide	*MC4R* agonism	Phase 3 trials in *POMC*/*LEPR* deficiency and Bardet–Biedl syndrome; phase 2 trial in acquired hypothalamic obesity	Marked weight reduction in genetically defined obesity	Mechanism-based treatment for selected monogenic obesity	Approved, indication-specific therapy (FDA, EC; status)	Only approved for genetically defined/syndromic indications and acquired hypothalamic obesity; contraindicated in *MC4R* deficiency and common polygenetic obesity	(Clément et al., 2020; Haqq et al., 2022; Roth et al., 2024; U.S. Food and Drug Administration, 2024; European Medicines Agency, 2026)
Liraglutide	GLP-1R agonism	Randomized clinical trials	Moderate weight reduction	Established anti-obesity pharmacotherapy	Established clinical therapy	Daily administration; gastrointestinal adverse effects	(Maki et al., 2023)
Orlistat	Gastrointestinal lipase inhibition	Established clinical use	Generally modest weight reduction	Peripheral, non-central mechanism	Established clinical therapy	Gastrointestinal adverse effects and adherence limitations	(Chakhtoura et al., 2023)
Phentermine/topiramate	Central appetite suppression through combined mechanisms	Established clinical trials	Moderate-to-substantial weight reduction	Effective oral pharmacotherapy	Established clinical therapy	Contraindications and central nervous system-related adverse effects	(Chakhtoura et al., 2023)
Naltrexone/bupropion	Modulates central appetite/reward pathways	Established clinical trials	Moderate weight reduction	Targets appetite and reward-related mechanisms	Established clinical therapy	Gastrointestinal and neuropsychiatric considerations	(Chakhtoura et al., 2023)

### 4.1 GLP-1 receptor agonists

The GLP-1 receptor agonist class has emerged as an important drug class for treating obesity, as these drugs affect appetite regulation, food intake, gastric emptying, and glucose metabolism simultaneously. STEP 1 showed that once-weekly semaglutide led to significant, clinically relevant weight loss in adults with overweight or obesity compared with placebo (Wilding et al., 2021). This effect results from sustained GLP-1 receptor activation, which increases satiety and lowers energy intake, making it an important molecular-targeted strategy for weight management. A systematic review and meta-analysis also found that GLP-1 receptor agonists led to significant weight loss and improved multiple cardiometabolic outcomes in non-diabetic people with obesity (Ansari et al., 2024).

GLP-1 receptor agonists have benefits beyond weight loss that affect clinically relevant cardiovascular outcomes. In the SELECT trial, semaglutide significantly lowered the risk of major adverse cardiovascular events (MACE) in adults with overweight or obesity and showed efficacy in cardiovascular disease (CVD) without diabetes, highlighting the potential for effective pharmacological weight management to also reduce cardiovascular risk (Lincoff et al., 2023). Overall, these results position GLP-1 receptor agonists as a class of drugs with significant weight-loss activity and other cardiometabolic benefits, and set the stage for the next-generation of incretin-based and multi-target drugs.

### 4.2 Dual and triple incretin-based therapies

New dual- and triple-incretin-based therapies have broadened the obesity drug landscape by combining different hormonal pathways to improve metabolic regulation and appetite suppression. The SURMOUNT-1 trial showed that in adults with obesity, dual GIP–GLP-1 receptor activation with tirzepatide produced significant, dose-dependent weight reduction, highlighting the therapeutic benefit of combined GIP–GLP-1 receptor activation (Jastreboff et al., 2022). Further progress has been made with the development of triple agonists, such as retatrutide, a combination of GIP, GLP-1, and glucagon receptor agonists, which produced significant weight loss in a phase 2 clinical trial (Jastreboff et al., 2023). These findings therefore support multi-receptor activation as a relevant molecular approach to achieve greater weight loss than single-pathway approaches. Importantly, tirzepatide represents an established clinical therapy for obesity, whereas retatrutide remains investigational; therefore, their shared multi-receptor mechanism should not be interpreted as equivalent clinical availability or as evidence that molecular profiling can currently determine which patients should receive one agent rather than another.

Comparative evidence also suggests that incretin polyagonists are a fast-growing field of obesity pharmacotherapy. The updated network meta-analysis showed significant variation in weight-loss effectiveness among drugs and suggested that multi-target drugs could provide greater treatment benefits (Xie et al., 2024). Dual and triple agonists provide broader pharmacological effects through simultaneous modulation of complementary metabolic pathways; however, their multi-target mechanism should not be interpreted as biomarker-guided or personalized treatment unless molecular characteristics are demonstrated to prospectively determine therapeutic selection or response. They represent a significant advance toward more effective, molecularly specific obesity treatments.

### 4.3 Other approved anti-obesity pharmacotherapies

Incretin-based therapies are not the only option for patients with contraindications, insufficient response, or intolerance; pharmacotherapies are clinically relevant and offer alternative mechanisms of action. Available medicines include orlistat, phentermine and topiramate, naltrexone and bupropion, liraglutide, and other medicines that act on the central or peripheral nervous system, with varying mechanisms of action, weight-loss results, side effects, and suitability for specific individuals. Beyond GLP-1 agents, several other drugs are in development to target gastrointestinal fat absorption, appetite-regulating neurotransmission, and central reward pathways (Chakhtoura et al., 2023). However, these therapies remain relevant in a comprehensive treatment approach, as obesity is a biologically diverse condition and not all patients respond equally to a single molecular pathway.

Choosing an anti-obesity drug must consider the drug’s likely weight-loss efficacy, comorbidities, cardiovascular risk, tolerability, contraindications, and the patient’s personal therapeutic profile. Existing pharmacotherapies can produce clinically significant reductions in body weight and related metabolic risk factors, and an appropriate choice of pharmacotherapy can reduce cardiovascular risk (Maki et al., 2023). For this reason, traditional non-incretin therapies remain relevant in molecularly targeted obesity treatment, as treatment should be tailored to the patient’s effectiveness, safety, comorbidities, and individual response.

Clinically, decision making for the selection of anti-obesity pharmacotherapy should be tailored to the severity of obesity, presence of obesity-related comorbidities, cardiovascular and metabolic risk, contraindications, tolerability and treatment preferences, as well as early response to treatment. Pharmacological therapies include GLP-1 receptor agonists and the GIP–GLP-1 co-agonist tirzepatide, which are established treatments for eligible patients, and other approved agents whose use depends on clinical factors, contraindications, cost, tolerability, or treatment response. In certain patients with genetically determined monogenic obesity or genetically determined syndromic obesity, genotype-directed therapy, such as setmelanotide, may be considered, following confirmation of the genetically indicated obesity. Conversely, new multi-agonists, molecular biomarkers, pharmacogenomic profiles or multi-omics-based approaches should be viewed as investigational and should not be offered as a typical precision-treatment suggestion.

### 4.4 Comparative efficacy, safety, and durability

The comparative effectiveness of anti-obesity pharmacotherapies depends on the molecules' mechanism of action, treatment intensity, and patient characteristics. A systematic review and meta-analysis suggest potential benefits of anti-obesity drugs for weight loss and cardiometabolic and psychological outcomes, but variability in efficacy and side-effect profiles may affect medication choice and tolerability (Liu et al., 2024). Weight-loss efficacy was significant in clinical trials, and a new meta-analysis showed tirzepatide was more effective than control at reducing body weight, without severe adverse effects (Qin et al., 2024). Interpret efficacy estimates from individual clinical trials with caution, as patient population, treatment duration, treatment dose, dose escalation schedule, and trial design may affect the weight loss observed. These results underline the need to consider the extent of weight loss, as well as safety, cardiometabolic effects, tolerability, and durability, when selecting drugs to treat obesity to support an individualized clinical approach to long-term obesity treatment, while molecularly informed treatment selection remains an emerging area requiring further validation. Table 2 presents a comparative overview of the principal anti-obesity pharmacotherapies, including their molecular mechanisms, clinical efficacy, therapeutic benefits, and major limitations.

## 5 Molecular determinants of therapeutic response

### 5.1 Genetic and epigenetic factors

Recognition of obesity and its hereditary links to relevant genes has been hindered by limited understanding of genetics at the human genome scale and the biological mechanisms implicated in obesity. Advances in genome-wide association studies (GWAS) and next-generation sequencing (NGS) have improved the identification of genetic associations and our understanding of both monogenic and polygenic factors contributing to obesity. GWAS have other genetic studies have identified many genes and genetic variants associated with obesity-related traits (Singh et al., 2017; Duis and Butler, 2022). Note that these estimates are not interchangeable: a locus is a region of the genome linked to a trait, a gene is a particular unit of the genome, and a variant is a particular change in the DNA. Obesity genetics can be divided into syndromic and non-syndromic obesity, with or without developmental delays and congenital abnormalities. For instance, early-onset obesity and developmental delay are linked to Prader-Willi, fragile X, Bardet-Biedl, Cohen, and Albright Hereditary Osteodystrophy (AHO) syndromes. Obesity that is not syndromic may have chromosomal, polygenic, or monogenic origins. Hence, genetic and epigenetic information should be considered in the context of its translational significance. Genetic variability could account for susceptibility to obesity or differences in treatment response, but genetic association does not necessarily constitute a genotype-based treatment plan. A clinically relevant molecular abnormality is known only when the connection to a relevant therapeutic approach is sufficiently established, as in some monogenic obesity syndromes.

This being a narrative review rather than a regional genetic association study, no new genetic or mutation data were generated from patients in the Ningbo/Zhejiang region. The genetic information presented here is therefore drawn from published literature, not necessarily estimates of genetic prevalence in a given region. For the study of Chinese and other East Asian populations, genetic studies are still very important to identify whether there are population-specific differences in genetic determinants of obesity and in response to drugs.

No patient level mutational analysis was done in the present narrative review; hence no new type of mutation or the frequency of mutations in specific regions can be reported. The literature describes pathogenic variants responsible for monogenic obesity, as well as common genetic variants associated with polygenic obesity susceptibility. Pathogenic variants in leptin-melanocortin pathway genes, such as LEP, LEPR, POMC, PCSK1, and MC4R, are clinically relevant monogenic defects. The clinical importance of these variants varies depending on the genetic disorder and the associated phenotype, and they may be either loss-of-function or impairing the signaling.

Genetic factors underlying obesity susceptibility must be differentiated from those underlying treatment response (pharmacogenetic). Emerging pharmacogenomic studies have found genetic variation contributes to variability in response to anti-obesity drugs, such as GLP-1 receptor agonists. However, associations have largely been small, inconsistent, and poorly reproduced across populations and treatment contexts. Other environmental or behavioral factors and the length of treatment may also interact with genetic effects and drug mechanism, and baseline metabolic phenotype. Thus, a genetic association is not a direct label for clinical utility in predicting treatment response.

Evidence on genotype-directed treatment in common obesity is less advanced than evidence for genotype-directed treatment in monogenic obesity. If associations with treatment response have been reported, the effect sizes are generally small compared to the overall range of weight loss, and the studies are few and far between. PRS can account for the aggregate heritability, but are only predictive if they are structured by population, phenotype definition, population calibration and the genetic architecture of the discovery datasets. This means that polygenic risk scores are not widely applicable at the individual-patient level to choose an anti-obesity treatment. Currently, no well-validated pharmacogenomic marker has been identified that is clinically useful for prediction and routinely used to inform the choice of approved pharmacotherapy for common polygenic obesity.

#### 5.1.1 Monogenic defects and targeted drugs

Monogenic obesity adheres to a Mendelian inheritance model, with individuals typically exhibiting extreme obesity resulting from mutations in a particular gene. Genetic alterations in the leptin–melanocortin pathway are mostly linked to monogenic obesity. In this route, leptin—an endocrine hormone from adipose tissue—attaches to the hypothalamus leptin receptor, prompting the synthesis of proopiomelanocortin (POMC). By cleaving POMC into melanocortin ligands such as α- and β-melanocyte-stimulating hormone, proprotein convertase subtilisin/kexin type 1 (PCSK1) accelerates binding and activation of the melanocortin-4 receptor (MC4R), decreasing food intake and increasing energy expenditure. Additionally, BDNF regulates this pathway (Koves and Roth, 2017). MC4R deficiency is currently the most common form of monogenic obesity, and loss-of-function variants in *MC4R* are reported in 2%–5% of patients with severe early-onset obesity (Hinney et al., 2022).

Clinically, the site of a monogenic defect dictates which drugs are used and how this treatment will affect the patient. Leptin dysfunction or congenital leptin deficiency can be treated with hormone supplementation. Administered subcutaneously at 0.03 mg/kg of lean body mass per day, human recombinant leptin (Metreleptin) significantly improves eating behaviour, decreases food intake and subsequently leads to weight loss. Furthermore, it was effective for the treatment of endocrine and metabolic disorders, such as central hypogonadism, hyperinsulinemia, hyperlipidaemia and hepatic steatosis (Hinney et al., 2022). Likewise, defects in POMC and *LEPR* can be pharmacologically corrected by activation of the melanocortin pathway downstream. Setmelanotide was shown to be beneficial in a phase II clinical trial for two patients with *POMC* deficiency (Kühnen et al., 2016). In phase III studies, 80% of the patients with *POMC* deficiency and about 45% of the patients with *LEPR* deficiency lost at least 10% of their body weight after about 1 year of treatment (Clément et al., 2020). Significantly, *POMC* deficiency and *LEPR* deficiency come before *MC4R,* meaning that setmelanotide can interact with an intact *MC4R*. By comparison, mutations in *MC4R* that cause the disease itself impact the receptor that setmelanotide acts on, so *MC4R* deficiency is not an approved disease indication for setmelanotide therapy. In the United States, setmelanotide has been approved by the FDA as a treatment for adults and children 2 years of age and older with obesity caused by Bardet–Biedl syndrome or biallelic mutations in *POMC*, *PCSK1* or *LEPR*, and since 2026 for adults and children 4 years of age and older with acquired hypothalamic obesity (Haqq et al., 2022; U.S. Food and Drug Administration, 2024). There is no approved indication for *MC4R* deficiency (U.S. Food and Drug Administration, 2024). The authorisation granted by the European Commission in the European Union includes adults and children ≥2 years of age with genetically confirmed Bardet–Biedl syndrome or biallelic loss-of-function deficiency of *POMC* (including *PCSK1*) or *LEPR* and, following its extension in 2026, adults and children ≥4 years of age with acquired hypothalamic obesity caused by hypothalamic injury or impairment (Roth et al., 2024; European Medicines Agency, 2026). *MC4R* deficiency is also not approved in the European Union (European Medicines Agency, 2026). At present, setmelanotide is in phase II or phase III clinical trials to target a number of other inherited defects within the *MC4R* pathway (Hinney et al., 2022).

#### 5.1.2 Polygenic obesity

Polygenetic obesity is the most common obesity type, which is affected by multiple genetic variants and environmental and behavioral factors, including stress, exercise, and diet (Tomiyama, 2018; Khera et al., 2019). GWAS have uncovered many loci associated with BMI, and the effect sizes of individual loci are typically small. Polygenic risk scores can be used to measure the genetic risk of obesity that is associated with the combined effects of obesity-associated genetic variants (Khera et al., 2019). The number of locations reported in various studies depends on the study population, sample size, obesity-related phenotype and definition of genetic associations, and hence reports of the number of loci should not be interpreted as exactly interchangeable estimates.

#### 5.1.3 Role of epigenetics in obesity development

One promising direction for future medical research is epigenetics, which may affect how people develop and treat illnesses (Moosavi and Ardekani, 2016). Epigenetics studies heritable changes in gene expression that cause phenotypic variation without altering the DNA sequence. Several factors can influence epigenetic modifications, including age, diet, environment, and illness. In particular body cells, epigenetics serves as a control layer to identify which genes are upregulated and which are downregulated (Al Aboud et al., 2018). DNA methylation, posttranslational alteration of histone proteins, and non-coding RNA (ncRNA)-associated gene silencing are currently recognized epigenetic processes. Environmental and dietary factors can affect the risk of obesity and diseases like type 2 diabetes. As a result, many researchers are interested in examining how various epigenetic changes may contribute to the development of these illnesses (Dendup et al., 2018).

### 5.2 Metabolic phenotypes and biomarkers

Biomarkers in this section can be separated by their biological and clinical use. Disease-associated biomarkers describe biological heterogeneity, metabolic dysfunction, inflammation, or disease severity, while predictive biomarkers are particularly linked to differential responsiveness to a specific treatment. Thus, identifying an obesity-related biomarker should not be regarded as proof of its usefulness for choosing a specific pharmacotherapy. Most of the biomarkers referred to in this section are, so far, used predominantly for biological characterisation or to generate hypotheses for treatment-response prediction but are not validated for treatment selection.

Obesity can be subclassified, which could help describe phenotypic and biological heterogeneity and propose hypotheses for personalised treatment. A study that subdivided obesity according to eating patterns identified two subgroups: hedonic eaters, who eat for the reward aspect of eating, and homeostatic eaters, who eat to control energy balance (Acosta et al., 2021). They considered energy expenditure (physical activity and resting energy expenditure), hedonic eating (anxiety and depression, and behavioural traits), and homeostatic eating (hunger, satiation, and satiety). They sorted people into four obesity phenotypes, for men and women respectively: “hungry brain,” “emotional hunger,” “hungry gut,” and “slow burn.” Based on these hypothesized mechanisms, rather than being established as routine treatment recommendations, the search for phenotype-matched pharmacotherapies was explored. One example is phentermine-topiramate for the “hungry brain” phenotype; another is bupropion-naltrexone for “emotional hunger,” liraglutide for “hungry gut,” and low-dose phentermine for strength training for “slow burn” (Acosta et al., 2021). In that study, the average weight loss was about 75% greater in the phenotype-guided group than in the group receiving standard pharmacological treatment (Acosta et al., 2021). These results, however, should be viewed as preliminary evidence for phenotype-based therapy rather than as a pharmacotherapy-selection algorithm. Replication is needed in ethnically and metabolically more diverse, multicentered populations, along with external validation and assessment of reproducibility across clinical settings. Phenotype-based approaches should be compared with current incretin-based therapies, such as GLP-1 receptor agonists and newer dual- or multi-agonist drugs, also, before these phenotypes can be used routinely for therapy selection.

Biomarkers are biological biomarkers of specific bodily conditions. Numerous biomarker categories were reported worldwide. MicroRNAs, inflammatory biomarkers, adipocytokines, oxidative stress, gut microbiota, nutritional levels, and blood cell profiles are the most commonly used. In this context, numerous obesity-associated or diagnostic biomarkers, such as inflammatory markers, adipocytokines, circulating microRNAs, hematological markers, and oxidative-stress markers have been reported. A few of these may be of prognostic or pharmacodynamic significance while others have been evaluated as possible predictive biomarkers of treatment response. They are not all ready for clinical use. To keep from over stating the strength of the evidence, the biomarkers reviewed are divided into three categories based on the available evidence. Exploratory markers are based on small studies, cross-sectional, retrospective or mechanistic studies and can be hypothesis-generating only. Validated markers are those that have been confirmed by prospective studies and/or independent, blinded replications in other studies, and are used to inform treatment decisions. Candidate (promising) markers are those that have been shown to be associated with treatment response in multiple studies, but have not been independently replicated or validated in a prospective study. These markers must be validated before they can be used in treatment decisions. For clinically actionable markers to be recognised, they must meet all of the following criteria: analytical validity (reliability and reproducibility of the assay); clinical validity (associates with the response to a specific intervention with a proven relationship in prospective clinical studies and independently reproduced); and clinical utility (demonstrates value of biomarker-guided therapy in improving clinical decision making and patient outcomes versus standard care). Therefore, a single study–even if designed carefully–is not enough for a marker to be clinically actionable. Selected monogenic obesity (Section 6.1) and routine clinical parameters (e.g., early treatment response, tolerability) are reviewed separately from molecular predictive markers.

*Inflammatory Biomarkers:* Researchers show that the obese population has inflammatory biomarkers (tumor necrosis factor, interleukin-6, and C-reactive protein) (Aleksandrova et al., 2017). These markers primarily reflect disease-associated inflammation and currently have limited evidence as validated predictive biomarkers for selecting anti-obesity pharmacotherapy.

- Micro-RNAs: Two miRNAs (miR-122 and miR-34a) were overexpressed in children with obesity and nonalcoholic fatty liver disease (NAFLD) and/or insulin resistance, while four miRNAs (miR-222, miR-142–3, miR-140-5p, and miR-143) were overexpressed in patients with obesity. Circulating miRNAs are promising diagnostic indicators for obesity and other conditions, including cardiovascular illnesses (Oses et al., 2019b). Their current role is primarily diagnostic or exploratory, and their value for predicting differential pharmacological response remains investigational.
- Adipocytokines: The apparent relationship between obesity and its related morbidities may be influenced by adipocytokines. It has a strong potential for predicting the presence of harmful cardiovascular diseases. Adiponectin, omentin, apelin, leptin, resistin, and fatty-acid-binding protein-4 appear to be useful obesity biomarkers, according to other reviews (Aleksandrova et al., 2017). Although adipocytokines may provide prognostic or biological information, their use as validated pharmacological treatment-selection biomarkers remains limited.”
- Blood Cells Profile: Platelet counts, plateletcrit (PCT) values, and platelet-to-lymphocyte ratio (PLR) values were all considerably higher in the morbid obese group. The obese population had higher, statistically significant white blood cell count and red cell distribution width (RDW) values (Erdal and İnanir, 2019). These measures are therefore better considered disease-associated or exploratory biomarkers rather than established predictors of response to specific anti-obesity drug

### 5.3 Predictors of treatment response and resistance

Predictors of treatment response are more relevant at a higher molecular level than biomarkers that simply describe obesity. A predictive biomarker should be analytically valid (a reliable, repeatable assay), clinically valid (a validated, independently replicated capacity to predict a clinically meaningful difference in response to a defined pharmaceutical intervention), and clinically useful (evidence that using the biomarker will improve clinical decision-making and outcomes). Therefore, candidate predictors described in the literature, such as hormonal, metabolic, inflammatory, genetic, metabolomic and early treatment-response markers, should be differentiated from validated treatment-selection markers. Correlations seen in exploratory or retrospective analyses may suggest that a drug has a potential predictor, but this does not mean the marker can be used effectively in clinical practice for therapeutic decision-making.

An increasingly innovative approach to treating obesity is using endogenous nutrient-stimulated hormones (incretins) produced by the gut. When nutrients enter the gut, intestinal hormones called incretins are released. These hormones stimulate insulin production and prevent glucagon release. Obesity impairs this reaction. Glucagon-like peptide-1 receptor agonists (GLP-1RAs) decrease hunger and food intake, slow stomach emptying, and improve glycemic control (Nauck and Meier, 2018b). In middle-aged adults with obesity or overweight and at least one weight-related complication, tirzepatide (Tz), a dual incretin that acts as both a GLP-1RA and GIP receptor agonist, had even greater effects on weight loss (−20.9%, or −22.1 kg, after a 72-week intervention vs. 3.1%, or −3.2 kg, placebo) (Jastreboff et al., 2022).

The initial weeks of treatment response, in the context of the medication and dose-escalation protocol, may be a helpful landmark for evaluation of efficacy and tolerability, and may guide dose optimization, continuation, medication switching, or behavioral/reinforcement support (Bays et al., 2022). Obesity-associated biomarkers may provide information relevant to treatment outcomes and help characterize biological heterogeneity and disease characteristics, while only a limited subset has been investigated as potential predictors of pharmacological response. Most currently lack sufficient prospective validation and clinical utility to guide routine treatment selection. In both pediatric and adult populations, leptin, adiponectin, and inflammatory markers such as cytokines have been shown to predict treatment outcomes, such as weight gain or dropouts, underscoring the need for targeted interventions (Zein et al., 2024). Fasting plasma ghrelin levels have been reported to have predictive value for response to liraglutide therapy in individual studies; however, such findings should be regarded as investigational until prospectively validated and shown to improve treatment selection in routine clinical practice, while caffeine metabolism-related metabolites have been found to predict greater weight-loss success in lifestyle interventions and pharmacotherapy, respectively (Aleksandrova et al., 2017). The major molecular and clinical determinants that may contribute to interindividual variation in response to anti-obesity pharmacotherapy are summarized in Table 3, with explicit distinction between clinically actionable factors, established genotype-directed treatment, and molecular determinants that remain investigational.

**TABLE 3:** Molecular and clinical determinants of response to anti-obesity pharmacotherapy.

Determinant	Biological basis	Potential influence on treatment response	Potential clinical application	Current clinical actionability	References
Monogenic obesity	Pathogenic variants can disrupt specific appetite- and energy-homeostasis pathways, particularly the leptin–melanocortin pathway	May produce pathway-specific treatment responses when the causal molecular defect is established	Selected patients may be screened by genetic testing for genotype-directed therapy, including setmelanotide for biallelic *POMC/PCSK1* or *LEPR* deficiency, and Bardet–Biedl syndrome; *MC4R* deficiency is not approved	Clinically actionable in selected genetically defined obesity	(Bhatnagar et al., 2025; Clément et al., 2020; U.S. Food and Drug Administration, 2024; European Medicines Agency, 2026)
Site of melanocortin-pathway defect (upstream of *MC4R* vs. *MC4R* itself	*MC4R* is a central regulator of appetite and energy balance downstream of *POMC* signaling	Upstream from MC4R (*POMC/PCSK1, LEPR)* defects predict response to *MC4R* agonism; pathogenic MC4R variants impair the drug target and do not	Treatment selection based on genotype for biallelic POMC/PCSK1 or LEPR deficiency; MC4R deficiency is not a rationale for using setmelanotide	Clinically actionable only for specific genetic indications; not established for common polygenic obesity	(Clément et al., 2020; U.S. Food and Drug Administration, 2024; European Medicines Agency, 2026)
Polygenic background	Multiple genetic variants contribute to obesity susceptibility and may contribute to interindividual variation in treatment response	May contribute to variability in weight loss and treatment response, but reported effects are generally modest and inconsistently replicated	Future pharmacogenomic stratification and risk prediction	Not currently clinically actionable for routine anti-obesity drug selection	(German et al., 2025)
Adipose cellular composition	Distinct adipocyte and stromal-cell populations have different metabolic functions and may contribute to differences in metabolic health	May contribute to biologically distinct obesity phenotypes and differences in therapeutic response	Molecular phenotyping and identification of candidate therapeutic targets or response subgroups	Investigational; not validated for routine treatment selection	(Reinisch et al., 2025)
Adipose transcriptomic profile	Tissue-specific gene-expression patterns reflect adipose metabolic function and dysfunction	May identify biologically distinct obesity phenotypes and molecular pathways associated with treatment response	Potential biomarker development and molecular stratification	Investigational; prospective clinical utility remains unvalidated	(Paulí et al., 2025)
Proteomic/metabolomic signatures	Altered proteins and metabolites reflect disrupted metabolic pathways and systemic metabolic phenotypes	May identify treatment-responsive phenotypes or candidate therapeutic targets	Multi-omics-based patient stratification and treatment-response prediction	Investigational; requires prospective validation before routine clinical use	(Li et al., 2025b)
Epigenetic alterations	Persistent molecular changes may remain after weight loss and may influence adipose biology and metabolic regulation	May contribute to weight regain, treatment resistance, and persistence of obesity-related biological changes	Identification of mechanisms underlying disease persistence and potential future treatment-response biomarkers	Investigational; not currently validated for treatment selection	(Hinte et al., 2024)
Gut-hormone signaling	Altered incretin and appetite-hormone signaling influences energy intake, satiety, glucose metabolism, and energy balance	May influence response to incretin-based pharmacotherapy and other pathway-targeted treatments	Mechanism-based selection of established therapies and development of future pathway-specific strategies	Molecular mechanism is clinically actionable through established drugs, but individual hormone profiles are not routinely validated for patient-specific drug selection	(Holst, 2024)
Leptin/adiponectin/inflammatory biomarkers	Reflect adipose-tissue function, systemic metabolic state, inflammation, and related biological heterogeneity	May be associated with treatment outcomes, treatment response, or treatment discontinuation	Biomarker-assisted treatment selection may be investigated in future studies	Not currently validated for routine pharmacotherapy selection	(Zein et al., 2024)
Early treatment response	Early changes in body weight or other clinical measures may indicate pharmacological sensitivity and treatment effectiveness	Poor early response may identify patients who require treatment reassessment or may have inadequate long-term response	Structured clinical reassessment, dose optimization, adherence assessment, or treatment switching	Clinically actionable as a clinical-response marker, but not a molecular precision biomarker	(Bays et al., 2022)
Baseline metabolic phenotype	Differences in glycemic, lipid, inflammatory, adipose, and cardiometabolic characteristics may influence treatment priorities and therapeutic benefit	May modify the relative benefits, risks, and therapeutic priorities of different pharmacotherapies	Individualized drug selection based on established clinical characteristics and comorbidities	Clinically actionable as part of conventional individualized treatment; not equivalent to validated molecular profiling	(Paulí et al., 2025)
Treatment tolerability	Gastrointestinal and other adverse effects influence treatment persistence and adherence	Poor tolerability may result in treatment discontinuation despite adequate efficacy	Dose titration, adverse-effect management, treatment switching, or combination strategies	Clinically actionable in routine treatment decisions	(Myerson and Paparodis, 2024)

## 6 Towards personalized pharmacotherapy for obesity

### 6.1 Biomarker-directed treatment selection

The best current clinical use of molecular guidance for choosing treatments is in the genetically defined obesity. Here, identifying a molecular defect in the pathogen can directly guide a targeted pharmacologic approach. One such pathway is the melanocortin pathway, which is especially important in selected types of monogenic obesity due to defects in *POMC/PCSK1* or *LEPR* (and in syndromic disease Bardet–Biedl syndrome).

This is a genotype-directed paradigm that should be differentiated from the common polygenetic obesity paradigm that is pharmacogenomics. Monogenic obesity has a pathogenic variant that can define the specific defective pathway which can be directly targeted by a specific treatment. In common obesity, genetic variation is spread across many genes and may reflect variation in susceptibility or response to treatment, rather than a single defect. Therefore, genotype-directed treatment of monogenic obesity is an example of precision therapy applied at the genome level, while pharmacogenomic prediction of response to conventional anti-obesity drugs is a new area of research.

The most common monogenic cause of severe early-onset obesity is loss-of-function variants in MC4R (Hinney et al., 2022), and over 150 variants, including frameshift, nonsense, and missense, have been described in this G protein-coupled receptor gene (Heyder et al., 2019). But the lack of MC4R is not a reason to prescribe setmelanotide. Setmelanotide is a drug that binds to MC4R. In the case of biallelic POMC (including PCSK1) or LEPR deficiency, the defect is upstream of a functioning receptor, meaning that activity at the receptor could be achieved through exogenous agonism as has been shown in single-arm open-label, multicentre phase 3 trials of subcutaneous once-daily setmelanotide in severe obesity due to POMC or LEPR deficiency (Clément et al., 2020). If the pathogenic variant involves the pharmacologic target (MC4R), the target is not working, and MC4R deficiency is not an FDA- or EMA-approved indication for setmelanotide in the United States or in the European Union (U.S. Food and Drug Administration, 2024; European Medicines Agency, 2026). Pathogenic MC4R variant alone should not therefore be a criterion for setmelanotide treatment. Preliminary data indicate that body weight decreases with incretin therapy (such as tirzepatide) in MC4R deficiency (Bhatnagar et al., 2025), suggesting that an effect outside the melanocortin receptor may still be at work. The genotype-directed approach in treating POMC/PCSK1 or LEPR deficiency should not be interpreted as a paradigm for pharmacogenomic prediction of obesity treatment response in common polygenic obesity, where clinical use of molecular markers has not yet been proven clinically useful for routine drug selection in obesity treatment.

### 6.2 Precision combination therapies

A few studies looked at the potential benefits of combining pharmacotherapy with other methods of managing weight loss, including drugs, lifestyle changes, and metabolic surgery. Other multi-agonists, such as Survodutide (a GLP1/glucagon co-agonist) and Cagrilintide (a long-acting amylin analogue), are being assessed in advanced clinical trials alongside current dual agonists (Kokkorakis et al., 2024; le Roux et al., 2024). These prospects offer a more comprehensive approach to improving efficacy and addressing the shortcomings of current medications, even though each agent targets distinct hormonal pathways. Although incretin-based treatments are effective, some patients may find subcutaneous delivery inconvenient.

Combining it with lifestyle management can also be beneficial. A study examined how exercise and liraglutide affected weight loss (Lundgren et al., 2021). The results showed that combining medication and exercise led to greater weight loss than either intervention alone. Additionally, post-treatment study showed that combination therapy increased weight loss maintenance following treatment discontinuation. One year after discontinuing treatment, a significantly higher percentage of patients (45%) who received both supervised exercise and medication maintained at least 10% weight loss than those who received only exercise (29%), only liraglutide (16%), or a placebo (10%) (Jensen et al., 2024).

### 6.3 Integrating molecular profiling into clinical practice

Clinical Decision-Support Systems: Electronic health records (EHRs) and clinical decision-support systems (CDSS) can provide doctors with effective, efficient point-of-care support. CDSS are digital tools that support practitioners at the point of care in promoting evidence-based weight-control guidelines. Designing tools that support comprehensive, evidence-based treatment choices for adult obesity may benefit health systems considering creating and implementing an obesity-focused CDSS (Fitch et al., 2023).

Role of Genetic Testing (GT) and Concerns: Based on the family history and presenting characteristics, genetic testing is chosen for severe early-onset obesity. Testing for both structural and sequencing variations should be prompted when clinical characteristics are present in addition to obesity. Evidence on how much a genetic variant can influence diagnosis, prognosis, or treatment determines the clinical use of GT (Oliveri et al., 2018).

Consumers, medical experts, and authorities often have concerns about GT. These issues, which affect both public and individual health, are not new. They involve ethics, law, psychology, and medicine. The commercialization and usage of these tests, as well as the storage of genetic data, raise ethical and legal concerns about autonomy, secrecy, privacy, and equity.

## 7 Emerging therapeutic strategies and future directions

### 7.1 Next-generation multi-agonists

Conventional anti-obesity drugs mostly target one route, like the GLP-1R. However, although synergistic effects can increase efficacy, it is unclear whether greater receptor complexity will improve tolerability or reduce treatment resistance.

Eli Lilly (Indianapolis, USA) developed the new triple hormone receptor agonist retatrutide. By activating the GLP-1R, retatrutide suppresses hunger and increases insulin secretion; by activating the GIP receptor, it increases insulin efficiency and improves energy utilization; and by activating the glucagon (GCG) receptor, it simultaneously enhances fat oxidation and energy consumption (Li et al., 2024). This multi-target mechanism could improve therapeutic response, but whether it reduces side effects or the likelihood of resistance development compared with multi-target versus single-target drugs remains unknown. Patients with T2D receiving 12 mg of retatrutide showed a 16.9% weight reduction (Jastreboff et al., 2023).

Novo Nordisk (Bagsvaerd, Denmark) developed a novel anti-obesity drug called amycretin. It targets both GLP-1 and the amylin receptor (AMYR). Subcutaneous injection and oral administration are the two dosage options that are currently being developed. Obesity is currently a suggested indication. With a tolerability profile comparable to GLP-1 and AMYR agonists, once-weekly subcutaneous amycretin at 60 mg produced a 24.3% weight reduction at week 36 in the phase 1b/2a study (Dahl et al., 2025).

### 7.2 Novel molecular targets

Rapidly developing molecular insights are uncovering new therapeutic targets, heralding next-generation drugs and multi-target regimens with increased durability and efficacy. GDF15 is a hormone in the transforming growth factor beta 1 (TGF-β) class. GDF15 suppresses appetite by activating canonical AKT, extracellular signal-regulated kinase (ERK), and phospholipase C (PLC) signalling pathways when it binds its specific receptor, GDNF family receptor α-like (GFRAL) (Yang et al., 2017). Moreover, GDF15-driven GFRAL activation maintains energy expenditure stability during calorie restriction by increasing muscle energy expenditure. The GDF15/GFRAL axis is a prime therapeutic target for obesity and associated diseases because of these metabolic regulatory roles (Wang et al., 2023). At present it is advisable to consider these emerging targets as investigational molecular strategies and not as proven precision-treatment targets. Likewise, genomic, epigenomic, transcriptomic, proteomic, metabolomic and cellular profiling could define phenotypes of obesity that are biologically distinct, or even be able to identify candidate subgroups of patients that could benefit from certain treatments, but this has yet to be validated in a prospective fashion and demonstrated to be clinically useful to select the most appropriate treatment. To maintain an obesity-specific translational framework, Figure 2 separates established molecularly targeted obesity pharmacotherapy from clinically actionable genotype-directed treatment for selected genetically defined obesity and from emerging investigational precision approaches. The latter include obesity-specific pharmacogenomic, biomarker, polygenic, and multi-omics strategies that remain under prospective evaluation.

**FIGURE 2:**
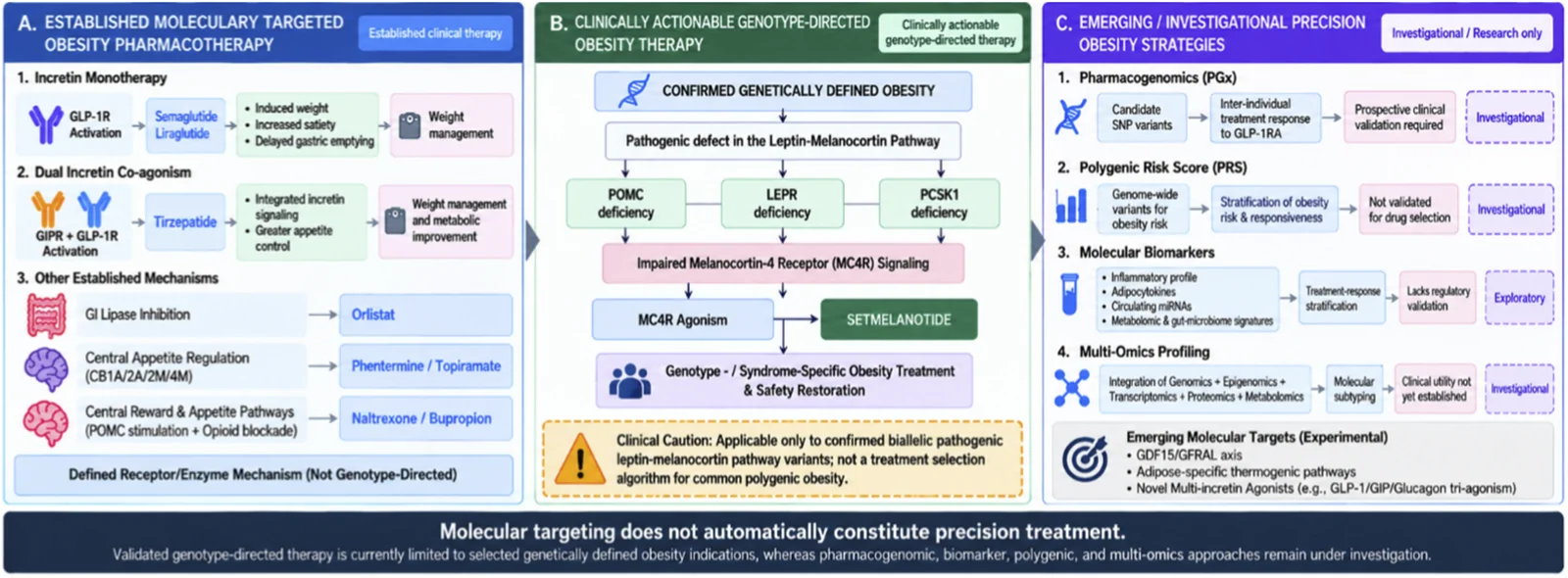
Current and Emerging molecularly guided pharmacotherapy for obesity. The figure highlights three levels of translatable molecularly guided obesity pharmacotherapy: **(A)** established obesity therapies targeting defined molecular pathways (e.g., GLP-1 receptor and GIP–GLP-1 receptor signaling); **(B)** clinically actionable genotype-directed therapy for selected genetically defined forms of obesity (e.g., melanocortin-pathway defects involving POMC, LEPR, and PCSK1 and treatment with the MC4R agonist setmelanotide); **(C)** and precision approaches that are emerging or investigational (e.g., pharmacogenomic response markers, polygenetic risk scores, biomarker-directed therapy, multi-omics profiling, and novel molecular targets). Targeting a pharmacotherapy at a molecular level is not the same as precision treatment of a patient. There is need to prospectively validate investigative, pharmacogenomic and multi-omics markers before they can be routinely used to inform anti-obesity pharmacotherapy selection.

### 7.3 Challenges in translating precision pharmacotherapy

Several hurdles arise when translating discoveries from omics research and emerging multi-agonist drug programmes into daily patient care. These encompass physiological tolerability, economic access and neurobehavioral reactivity to treatment.

Dose-Escalation Toxicity and Physiological Barriers: Gastrointestinal (GI) side effects are the most common cause of treatment discontinuation, both in clinical trials and in clinical practice (Ismaiel et al., 2025). GI intolerance was a major driver for patients and physicians to discontinue GLP-1 receptor agonists (GLP-1 RAs) in real-world clinical practice, and the relative burden varied between physicians and patients in the largest cross-sectional survey to date. Inadequate glycemic control (45.6%), nausea or vomiting (43.8%), and other GI adverse effects (36.8%) were the most frequent reasons for discontinuation reported by surveyed physicians (n = 443). The most frequent complaints were nausea (64.4%) and vomiting (45.4%) among the patients surveyed (Sikirica et al., 2017).

Economic Barriers: The cost of GLP-1 RA therapy, and whether patients or healthcare systems cover it, affects therapy availability and adherence. The cost of a 30-day treatment with subcutaneous semaglutide was estimated at USD 40, assuming standard manufacturing conditions, which differs from the list, negotiated, reimbursed, or out-of-pocket price (Levi et al., 2023). Real prices depend on manufacturer pricing, procurement and negotiation requirements, insurance coverage, reimbursement criteria, taxation, and patent/market-exclusivity conditions, and differ considerably between countries and healthcare systems. This figure should therefore not be seen as the price to patients or health systems, or as a direct measure of the drug’s affordability.

## 8 Conclusion

The paradigm shift in obesity pharmacology is from broad appetite suppression to a more targeted approach to interrelated biological pathways. The data presented in this article show that obesity is a significant source of molecular heterogeneity, including hypothalamic circuits, gut–brain hormonal signalling, adipose-tissue dysfunction and inflammation, genetic variation, and long-lasting epigenetic changes. This diversity offers a mechanistic explanation for the variations in the therapeutic efficacy and tolerability, weight regain and long-term response. GLP-1 receptor agonists and GIP–GLP-1 co-agonism have demonstrated clinical utility in obesity pharmacotherapy, whereas glucagon-containing multi-agonists and other emerging receptor combinations remain investigational despite promising clinical-trial results. These are just some of the novel targets emerging in the melanocortin, amylin, central neural, and adipose sectors, and each offers the potential for new treatment approaches.

However, molecularly guided pharmacotherapy for obesity is currently less broadly used than one might think from the very general name of precision obesity medicine. GLP-1 receptor agonism, GIP–GLP-1 co-agonism, and other receptor-targeting treatments can be used in the clinical care of obesity, and genotype-directed therapy is clinically relevant for specific syndromic or monogenic forms of obesity. Most biomarker-guided, pharmacogenomic, molecular-phenotyping, and multi-omics approaches remain investigational. These strategies may ultimately drive more efficient treatment choice, combination therapy, and overcoming therapeutic resistance; however, prospective validation, population-level reproducibility, and a proof of improved patient outcomes following molecular stratification over current clinical decision-making is required.

Molecularly guided pharmacotherapy, then, should be considered one element in a spectrum of evidence, not a single treatment paradigm. Existing and emerging drugs, the characterization of disease-associated biomarkers, and selected genetic defects may directly influence treatment in certain obesity syndromes, all of which are explained by molecular mechanisms. However, most proposed predictive biomarkers, pharmacogenomic signatures, and multi-omics profiles remain insufficiently validated for general use in treatment selection. Future prospective studies are needed to determine whether molecular stratification can be reproducibly used to identify patients most likely to benefit from a particular therapy and whether this approach can deliver better clinical outcomes than current clinical approaches.
